# Observations and hypotheses for potential generational differences in cardiometabolic risk factors in U.S. South Asians

**DOI:** 10.1016/j.ihj.2021.10.014

**Published:** 2021-11-01

**Authors:** Trishna Parikh, Anjitha Saji, Aayush Visaria

**Affiliations:** McGovern Medical School, University of Texas Health Science Center, Houston, TX, USA; McGovern Medical School, University of Texas Health Science Center, Houston, TX, USA; Department of Medicine, Rutgers Robert Wood Johnson Medical School, New Brunswick, NJ, USA

**Keywords:** Epidemiology, South Asian, Cardiovascular risk factors, BMI, Obesity

## 1 To the editor

We read with pleasure the work of Shah et al titled “Cardiovascular health and subclinical atherosclerosis in second generation South Asian Americans: The MASALA study”, which found no significant difference in cardiometabolic risk factors between first- (G1) and second-generation (G2) South Asian (SA) Americans.^1^ Despite these negative findings, we would like to point out observations from the presented data as insignificant differences were likely due to low statistical power and lack of normality for parametric tests/linear regression.

There seem to be increasing trends in BMI (26.1, 27.0, 27.6 kg/m^2^), total cholesterol (190, 204, 199 mg/dl), and triglycerides (135, 154, 163 mg/dl) from late-G1 to intermediate generation (G1.5) immigrants to G2 SAs. This is in line with hypotheses suggesting adoption of Western diets and acculturation has increased prevalence of obesity and dyslipidemia in SAs. Accordingly, coronary artery calcium was also present in a greater proportion of G2 than G1 participants. However, several participants were taking statins, so analyses among those not taking cholesterol-lowering medications and in a greater number of G2 participants would be needed.

A few foreign studies have shown that the health landscape of G1 and G2 South Asians are different. In Ontario, Canada, immigrants had differing prevalence of diabetes when compared to a “non-immigrant” population.^2^ In the UK, there were generational differences in diabetes, BMI, and asthma prevalence among South Asians.^3^ This suggests potential SA heterogeneity across generations, further complicating an already culturally heterogeneous population.

Future studies should capture sociocultural, psychological, and clinical factors.^⁴^ There are far reaching implications of U.S. generational differences as preventive screening and diagnostic criteria may need to reflect changes in disease risk. If there is increased risk of cardiovascular disease in G2 SAs, it is of paramount importance to intervene as soon as possible as the number of G2 SAs increases rapidly.

## Ethics approval and consent to participate

This study was exempt from Rutgers's Institutional Review Board approval.

## Funding

No funding provided for this study.

## Declaration of competing interest

The authors declare that they have no competing interests.
